# Neural networks and econometric models: Advancing brain connectivity for Alzheimer’s drug development

**DOI:** 10.4103/NRR.NRR-D-25-00317

**Published:** 2025-06-19

**Authors:** Lorenzo Pini, Paolo Pigato, Gloria Menegaz, Ilaria Boscolo Galazzo

**Affiliations:** Padova Neuroscience Center, University of Padova, Padova, Italy; Department of Neuroscience, University of Padova, Padova, Italy; Department of Economics and Finance, University of Rome Tor Vergata, Rome, Italy; Department of Engineering for Innovation Medicine, University of Verona, Verona, Italy

Advances in Alzheimer’s disease (AD) research have deepened our understanding, yet the mechanisms driving its progression remain unclear. Although a range of *in vivo* biomarkers is now available (e.g., measurements of amyloid-beta (Aβ) and tau accumulation – the molecular hallmarks of AD – structural magnetic resonance imaging (MRI), assessments of brain metabolism, and, more recently, blood-based markers), a definitive diagnosis of AD continues to be challenging. For example, Frisoni et al. (2022) proposed a shift from a deterministic, amyloid-centered approach to a probabilistic framework that integrates genetic and environmental factors. Similarly, Dubois et al. (2024) cautioned against diagnosing AD based solely on molecular markers in cognitively normal individuals, advocating instead for the designation of “at risk” individuals. These perspectives reflect an evolving understanding of AD that is continuously reshaping both clinical and pharmacological approaches. Moreover, the recent approval of two disease-modifying agents (Lecanemab and Donanemab) that target misfolded Aβ proteins has underscored significant limitations, particularly their moderate impact on clinical and cognitive outcomes. The discrepancy between the improvements in AD biomarkers with anti-Aβ drugs and their limited clinical benefits underscores the need for a new paradigm. In this context, assessing Aβ levels can be compared to measuring blood pressure: just as high blood pressure does not inevitably lead to cardiovascular disease, and some individuals with the disease may not have elevated blood pressure, the multifactorial nature of AD suggests that Aβ accumulation alone does not define the disease.

Within this complex landscape, brain connectivity is increasingly recognized as a pivotal factor with the potential to reshape our understanding of AD. Evaluating the hierarchical organization of the brain could provide a novel perspective on AD pathophysiology. Brain connectivity encompasses both structural and functional dimensions. The former refers to the anatomical pathways linking different brain regions, primarily measured through diffusion MRI-based tractography. Functional connectivity captures temporal correlations in neural activity between distant brain regions, typically assessed via resting-state fMRI. In AD, disruptions in large-scale brain networks, such as the default mode network, a set of brain regions encompassing the posterior cingulate cortex, inferior parietal lobe, medial temporal cortex, and medial prefrontal cortex, are well-documented and correlate with disease progression and cognitive decline (Vogel et al., 2023). Recent studies showed a substantial overlap between the spatial distribution of Aβ and the topography of the default mode network. At the same time, tau accumulates preferentially in regions closely connected to tau epicenters (specific brain regions where tau pathology first emerges) and spreads from there along brain connectivity pathways, reflecting its transmission through neural networks (Vogel et al., 2023). A deep understanding of mechanisms linking protein propagation and connectivity alterations could offer critical insights into AD therapeutic strategies, shaping an integrative and effective approach to diagnosis and treatment.

We have recently proposed that brain connectivity measures could hold substantial value in pharmacology, as surrogate markers of drug efficacy, as enrichment strategies for clinical trials, and as tools for guiding innovative therapeutic approaches (Pini et al., 2024). Indeed, connectivity alterations do not follow linear alterations in AD, and more in general in neurological diseases,(Zhang and Pini, 2024) suggesting that pathophysiological alterations are not just incremental, but rather complex interplay in which different factors are at play. Notably, non-linear brain connectivity alterations appear to predict cognitive and clinical outcomes, with hyper-connectivity potentially reflecting compensatory responses, while hypo-connectivity patterns are associated with cognitive impairment and scale with clinical severity (Pini et al., 2024). Integrating connectivity-based biomarkers with multimodal neuroimaging and deep learning approaches may enhance the predictive accuracy of disease progression and treatment response. This perspective underscores the need for a paradigm shift, moving beyond a solely pathological viewpoint to a network-based understanding of AD, where disruptions in connectivity are seen not just as consequences but as potential drivers of disease dynamics. Brain connectivity measures could contribute to the development of novel disease-modifying therapies, serving as surrogate markers of efficacy, particularly in relation to cognitive outcomes. Functional connectivity fingerprints may help predict which patients are most likely to benefit from a given treatment, offering insights into inter-individual variability in therapeutic response. Unlike molecular and degenerative markers, which provide a static representation of pathology, brain connectivity reflects dynamic mechanisms that underlie neural reserve, compensation, and maintenance, processes that may mitigate the effects of neurodegeneration. As such, connectivity outcomes could be particularly valuable in the context of clinical trials, offering a means to identify patient subgroups that might respond differently to specific interventions. A similar paradigm shift has been recently proposed in glioblastoma research (Salvalaggio et al., 2024), highlighting the broader clinical relevance of connectivity-based approaches in neurology. However, a critical point regards the way brain connectivity is analyzed. While most of the present literature relies on univariate analysis, assessing which network/regions are altered across groups of patients, the analysis of the brain connectome could be significantly enhanced by leveraging recent advancements in deep learning and models from different disciplines, such as econometrics. Here, we discuss these two potentials “*Use Cases*” in the context of AD, highlighting how they could be integrated into pharmacological research to refine therapeutic strategies and improve treatment outcomes.

Given the high-dimensional and multivariate nature of brain connectivity data, novel deep learning models are crucial for advancing the pharmacological field. Such approaches could unravel complex interactions among brain networks and pathological markers, promoting personalized therapeutic strategies. While deep learning algorithms have demonstrated excellent performance in classification and prediction tasks, even in complex tasks such as distinguishing between Aβ-positive and Aβ-negative individuals, they are frequently criticized for their lack of transparency (“black-box models”) and interpretability. To address this limitation, eXplainable Artificial Intelligence methods have been proposed, allowing us to identify how the input features contribute to the final (model) outcome. Widely used eXplainable Artificial Intelligence methods, such as attention mechanisms, layer-wise relevance propagation, Shapley additive explanations, and local interpretable model-agnostic explanations, can help shed light on the underlying decision-making processes and identify the most relevant features. An example of a multimodal and explainable deep learning-based framework for the classification of Aβ status in the AD continuum, exploiting anatomical and connectivity MRI-based information, has been recently proposed (Dolci et al., 2025). Good classification performance has been shown and, at the same time, those brain areas mostly contributing to the final decision, such as hippocampus, thalamus, and precuneus, were highlighted. By improving interpretability, eXplainable Artificial Intelligence can enhance their applicability in clinical and pharmacological settings, such as identifying potential brain targets, and bridging the gap between complex computational approaches and real-world medical decision-making. While deep learning models have significantly advanced the study of brain connectivity in AD, offering more precise, data-driven insights into disease mechanisms, their primary application has largely been in distinguishing AD patients from controls at various clinical and prodromal stages. However, their potential reaches far beyond classification. A particularly promising application is in predicting individual responses to different pharmacological interventions, enabling the identification of both good and poor responders. Effectively treating complex disorders like AD requires a more comprehensive approach, one that integrates multiple targets and pathways rather than focusing on isolated mechanisms. These advancements highlight the transformative potential of artificial intelligence-driven brain connectivity analyses, not only in enhancing diagnostic accuracy but also in revolutionizing pharmacological approaches through personalized, system-based treatment strategies.

While deep learning excels at capturing complex, high-dimensional relationships in neuroimaging and clinical data, econometric volatility models could offer a complementary framework for modeling time-dependent fluctuations in functional brain signals. These approaches, though methodologically distinct, share a common goal: identifying latent patterns in dynamic systems that uncover hidden structures within large, multivariate datasets. Bridging deep learning and econometric modeling could thus enhance predictive accuracy in AD research, particularly when integrated within multimodal frameworks. Several econometrics models have proved fruitful in the analysis of time series in economics and finance. Recently, Dugo et al. (2024) proposed modeling market volatilities (quantitative measures of price fluctuations over time) using a multivariate fractal model that may mimic neural signal time-series data. This model effectively captures the complex dynamics of volatility time series across various financial indices, in particular regarding their strong cross-correlations and the asymmetries in their cross-covariance structures, as demonstrated with data from the Oxford-Man Realized Library. Interestingly, similar characteristics can be observed in neural time-series characterized by complex, non-linear fluctuations, and strong inter-connections, analogous to characteristics observed in financial markets. Pathological and compensatory AD mechanisms can alter these complex interactions. Applying these econometrics models to brain connectivity could provide a novel framework for understanding how the brain’s neural networks evolve over time, particularly in response to the AD pathophysiological cascade. The application of econometric models to brain connectivity could also transform AD drug development strategies. Traditional drug discovery has often relied on the “one disease, one target, one drug” paradigm, where drugs are designed to target a specific molecular pathway. However, AD is a heterogeneous disease, and single-target strategies often fail to produce significant clinical benefits (Hopkins, 2008). In contrast, multi-target strategies that account for the complexity of the disease and its underlying brain network disruptions are gaining traction. Just as financial markets require multi-dimensional models to understand price fluctuations across different assets and indices, AD treatment strategies may benefit from integrating multivariate models of brain connectivity. Specifically, the application of suitable econometric models has the potential to allow for more precise matching of patients to specific therapies, improving the efficacy of clinical trials and reducing the costs and risks associated with drug development. The application of volatility models to brain connectivity is a clear example of how interdisciplinary collaboration can advance our understanding of AD and drug development. Just as economists have developed complex algorithms to predict market trends, similar approaches can be applied to the study of brain networks. The challenge lies in adopting a highly multidisciplinary approach, where expertise from fields like pharmacology, neuroscience, mathematical economics, and econometrics converge to develop innovative models for understanding brain dynamics and therapeutic response. By applying these cross-disciplinary methods, we can drive a paradigm shift in AD research, transforming the way we think about diagnosis, progression, and treatment, with the ultimate goal of developing more effective, personalized therapies.

Finally, brain connectivity has the potential to drive drug repositioning, the strategy of identifying new therapeutic applications for existing drugs. The growing integration of deep learning into biomedical research is reshaping drug discovery and development. Drug development remains a time-consuming and costly endeavor, with many new therapeutics requiring extensive testing and optimization before reaching clinical application. Drug repurposing, identifying new therapeutic applications for existing drugs, addresses this challenge by leveraging real-world data and computational methods to accelerate the discovery process. Deep learning algorithms, applied to large-scale clinical and epidemiological datasets, can generate new hypotheses for drug efficacy by identifying patterns in patient outcomes. High-throughput computational approaches are now enabling the rapid screening of multiple drug candidates simultaneously, moving beyond traditional hypothesis-driven methods. These models, applied to real data recently highlighted how five drugs (pantoprazole, gabapentin, atorvastatin, fluticasone, and omeprazole) originally intended for other indications with potential benefits for AD (Zang et al., 2023). These advancements, combined with connectivity-based insights, could transform pharmacological strategies by facilitating more precise and personalized treatment approaches while also expanding the scope of available therapeutic options.

**Figure 1:**
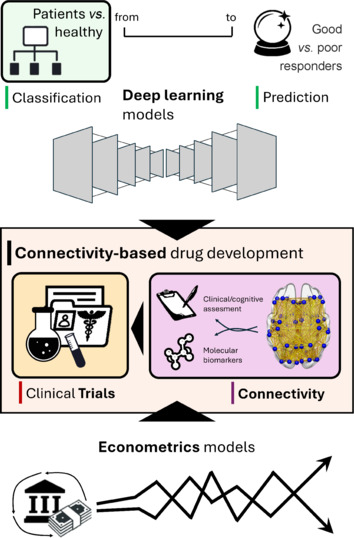
Integrating brain connectivity, deep learning, and economic models into Alzheimer’s pharmacological research. Brain connectivity measures could be incorporated into pharmacological research to better understand drug effects on neural circuits by examining the relationship between connectivity, cognitive/clinical outcomes, and misfolded protein accumulation (central panels). The analysis of brain connectivity should leverage recent advances in deep learning, particularly in distinguishing potential responders from non-responders to pharmacological treatments (top panels). Furthermore, novel models derived from economic mathematics should be explored as alternative methodologies to analyze connectivity data, potentially offering new perspectives on drug response prediction and patient stratification (bottom panel). Created with GIMP software.

In conclusion, we advocate for incorporating technological advancements to refine the role of brain connectivity measures in drug development and therapeutic strategies (**Figure 1**). While deep learning and econometrics models offer powerful predictive tools, the inclusion of dynamic functional connectivity is crucial to capture time-varying neural processes that may underlie cognitive resilience and disease progression and should be considered in such frameworks. Unlike traditional static connectivity, which assumes stable interactions between brain regions, dynamic functional connectivity tracks fluctuations in connectivity over time, providing insights into network reconfigurations that may distinguish adaptive from pathological changes. Furthermore, leveraging multi-omics data, including genomics, transcriptomics, and metabolomics, can enhance precision medicine by linking molecular mechanisms to connectivity alterations. A truly comprehensive framework should not only refine the role of brain connectivity measures in drug development but also embrace the complexity of AD as a network-based and multi-system disorder, ultimately guiding personalized therapeutic strategies.
